# Supplementary material for: Nanobody‐mediated complement activation to kill HIV‐infected cells
Source: EMBO Mol Med. 2023 Feb 17;15(4):e16422. doi: 10.15252/emmm.202216422 (PMC10086584; doi:10.15252/emmm.202216422)
Supplement: Supplementary file 1 — Expanded View Figures PDF [file EMMM-15-e16422-s001.pdf]

Expanded View Figures

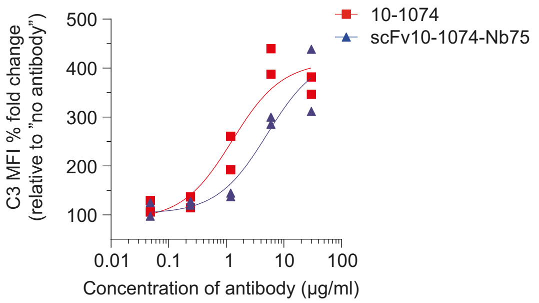

Figure EV1. Concentration-dependent C3 deposition of infected primary CD4 T cells.

Primary CD4 T cells infected with HIV-1$_{NL4\text{-}3\text{-}eGFP}$ were incubated with NHS and the indicated concentrations of antibodies or BiCEs. After 24 h, surface level of C3 was measured by flow cytometry. *n* = 2 donors.

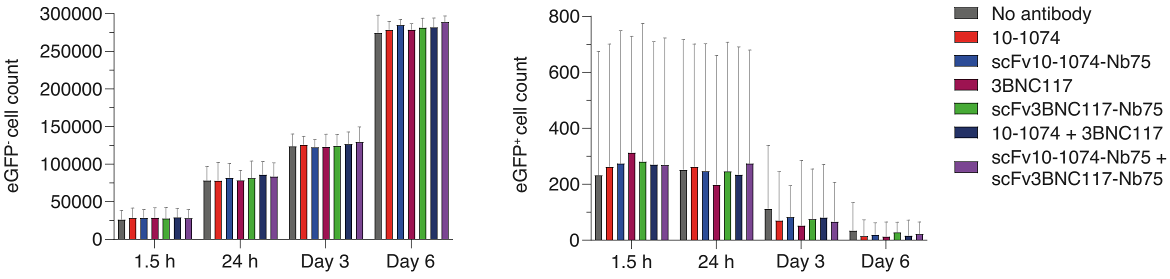

Figure EV2. Presence of NHS and anti-HIV antibodies or BiCEs do not affect uninfected cells.

Primary CD4 T cells infected with HIV-1$_{NL4\text{-}3\text{-}eGFP}$ and uninfected CD4 T cells were incubated with NHS and the indicated antibodies or BiCEs in presence of an HIV-inhibitor. The cell count was assessed by flow cytometry at the indicated time points. *n* = 4 donors. Data are presented as median with interquartile range.

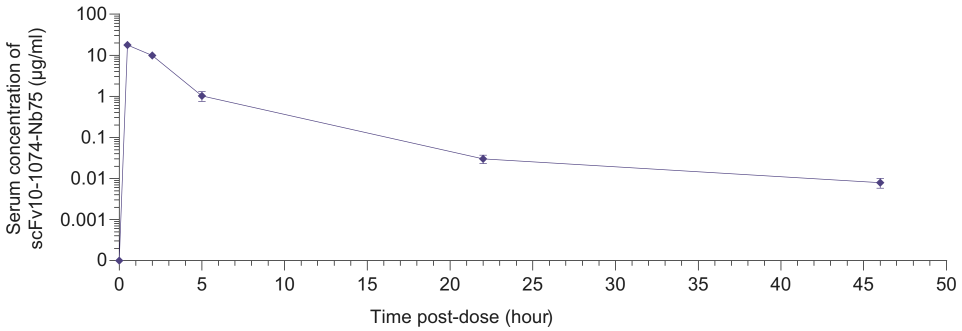

Figure EV3. Pharmacokinetic study of BiCE.

ScFv10-1074-Nb75 was administered intraperitoneally (I.P.) at 10 mg/kg mouse. Blood was collected from the tail vein at the indicated time points. The serum concentration of scFv10-1074-Nb75 was analyzed using a time-resolved immunofluorometric assay (TRIFMA). *n* = 4 mice. Data are presented as mean ± SD.

© 2023 The Authors

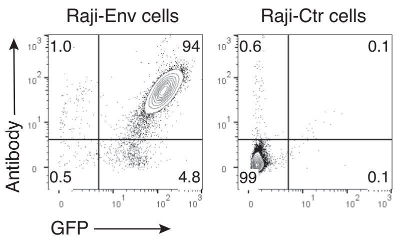

Figure EV4. Co-expression of GFP and Env on Raji cells.

Raji cells expressing HIV-1_{YU-2} envelope (Raji-Env) or Raji control cells (Raji-Ctr) were incubated with anti-Env monoclonal antibody 10-1074. After 45 min, binding of 10-1074 to HIV-Env on cells was assessed by flow cytometry using a BV421-conjugated anti-IgG Fc antibody. The numbers indicate the percentage of cells.

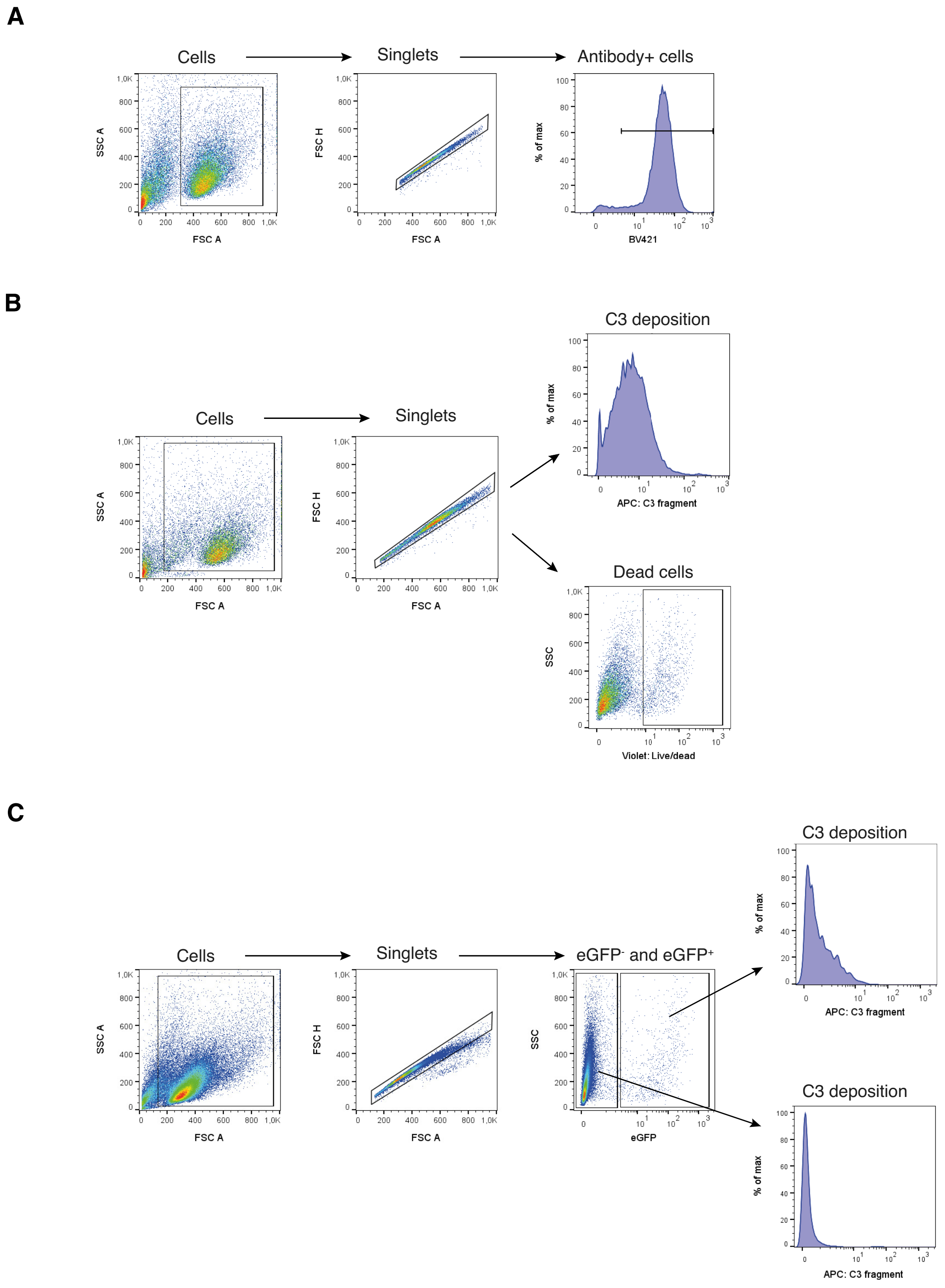

Figure EV5. Gating strategy for flow cytometry assays.

A Gating strategy for anti-HIV antibody surface staining of Raji cells. BV421-conjugated anti-IgG Fc or BV421-conjugated streptavidin were used to discriminate cells with bound anti-HIV antibody.

B Gating strategy for complement activation assays of Raji cells. APC-conjugated anti-C3 antibody and zombie violet live/dead marker were used to differentiate cells with deposited C3 and dead cells, respectively.

C Gating strategy for complement activation assays of primary CD4 T cells. EGFP expression was used to distinguish HIV-infected cells. APC-conjugated anti-C3 antibody was used to discriminate cells with deposited C3.

 © 2023 The Authors
