# Supplementary material for: Nanobody‐mediated complement activation to kill HIV‐infected cells
Source: EMBO Mol Med. 2023 Feb 17;15(4):e16422. doi: 10.15252/emmm.202216422 (PMC10086584; doi:10.15252/emmm.202216422)
Supplement: Supplementary file 3 [file EMMM-15-e16422-s002.pdf]

EMBO Molecular Medicine

Nanobody-mediated Complement Activation to Kill HIV-infected Cells

Maria Pedersen, Dennis Pedersen, Mikael Winkler, Heidi Olesen, Ole Søgaard, Lars Østergaard, Nick Laursen, Anna Rahimic, and Martin Tolstrup
DOI: 10.15252/emmm.202216422

Corresponding author: Martin Tolstrup (mtol@clin.au.dk)

Review Timeline:

Submission Date:	8th Jun 22
Editorial Decision:	4th Jul 22
Revision Received:	6th Jan 23
Editorial Decision:	24th Jan 23
Revision Received:	2nd Feb 23
Accepted:	3rd Feb 23

Editor: Zeljko Durdevic

Transaction Report:

(Note: With the exception of the correction of typographical or spelling errors that could be a source of ambiguity, letters and reports are not edited. Depending on transfer agreements, referee reports obtained elsewhere may or may not be included in this compilation. Referee reports are anonymous unless the Referee chooses to sign their reports.)

1st Editorial Decision 4th Jul 2022

Dear Dr. Tolstrup,

Thank you for the submission of your manuscript to EMBO Molecular Medicine. We have now received feedback from the three reviewers who agreed to evaluate your manuscript. As you will see from the reports below, the referees acknowledge the interest of the study but also raise important and partially overlapping concerns that should be addressed in a major revision. We would appreciate in vivo experiments as suggested by the referees #2 and #3, however, they are not essential. That being said, biodisponibility and pharmacokinetics experiments should be provided.

Further consideration of a revision that addresses reviewers' concerns in full will entail a second round of review. EMBO Molecular Medicine encourages a single round of revision only and therefore, acceptance or rejection of the manuscript will depend on the completeness of your responses included in the next, final version of the manuscript. For this reason, and to save you from any frustrations in the end, I would strongly advise against returning an incomplete revision.

We would welcome the submission of a revised version within six months for further consideration. Please let us know if you require longer to complete the revision. I look forward to receiving your revised manuscript.

Yours sincerely,

Zeljko Durdevic
Editor
EMBO Molecular Medicine

***** Reviewer's comments *****

Referee #1 (Remarks for Author):

In this manuscript, Lange-Pedersen and colleagues report the creation and the validation of complement-activating anti-HIV-1 nanobodies. These new molecules attach to HIV-1 env on one side (using fragments from very well-known and potent antibodies) and to C1q on the other side. They call these molecules bispecific complement engager (BiCE). Then, they validate their capacity to mediate complement-mediated cytotoxicity in two different systems. First, using a cell line ectopically expressing HIV-1 Env and second using infected cells. They observe that their BiCE bind, trigger complement deposition and kill target cells. However, the outcome differs between the models, with either a rapid cell lysis in the cell line, or a delay killing in CD4 T cells. Pros: the BiCE platform is innovative and interesting, the authors successfully demonstrate the capacity of this platform to engage C1q and subsequently the complement cascade. Cons: the intrinsic resistance of HIV-1-infected cells limit the capacity of their compound to eliminate infected cells, it seems that BiCE performed just as well as antibodies to elicit complement, a few controls are missing. Overall, this is an interesting report, which deserves a few additional experiments and analysis prior to its publication.

Specific comments:

-The various compounds are compared on a scale in ug/mL. Given their likely various molecular mass, it would be much more appropriate and infortive to compare them using molarity. It should be done prior to publication.

- In figure 2A, additional compounds should be tested: the nanobody without the C1q binding part, and antibody fab, the C1q bind part alone.. Overall, the anti-HIV-1 capacity of each individual part of BiCE deserves a more comprehensive assessment (of course the appropriate controls must be including). Minor point : the graph could be split into 2 graphs, according to the "backbone" either 3BNC117 or 10-1074, to facilitate reading.

- In the experiment with infected cells the choice of the viral strain is surprising and controls are lacking. Indeed, NL4.3 is known to be insensitive to 10-1074 neutralization and ADCC. Thus, it is expected to observe a lack of activity of the 10-1074-based BiCE. This is not the case in the author's data, which is possible, but additional controls are needed to support this claim. Indeed, there is neither an isotype control antibody nor an irrelevant BiCE included as controls in the experiment. It should be performed to ensure the relevance of the observed phenotype. Measuring the binding capacity of the BiCE at the surface of infected cells could also help interpret the data.

-In figure 2 the bonding could be analyzed using the MFI, as the % of positive cells appears saturated in almost all conditions.

-Figure 1 C left, colors are uneasy to read

-in the discussion, the sentence "Moreover, the smaller size of BiCEs may allow competition for epitope sites in proximity, such as the V3 loop and CD4 binding sites of HIV Env (Labrijn et al, 2003). " is confusing, as 10-1074 and 3BNC117 target these epitopes and work very efficiently in combination.

Referee #2 (Comments on Novelty/Model System for Author):

In general, the work is methodologically correct. These in-vitro and ex-vivo results are promising and it would be interesting to see how these therapeutic tools perform in preclinical settings; this will aid to better understand PKs/ biodistribution of these constructs and in vivo performance. Further, in vivo testing will inform if the absence of Fc triggering and reduced neutralizing activity observed for BiCEs (compared to full parental NAbs) have an impact on in vivo efficacy

Referee #2 (Remarks for Author):

In the present manuscript entitled "Nanobody-mediated Complement Activation to Kill HIV-infected Cells", Maria Lange Pedersen and colleagues describe the development and preliminary in vitro and ex vivo testing of a novel immunotherapy tool designed for HIV-1 treatment. This approach consists in a bispecific molecule directed to HIV-1 Env (single-chain variable fragments of broadly neutralizing antibody 3BNC117/ 10-1074) and the complement-initiating protein C1q (C1qNb75 nanobody). The constructs were tested for virus neutralization, HIV-Env recognition and complement activation in YU-2 Env expressing cell line. Furthermore, the authors tested the ability of anti-HIV BiCE to induce complement activation and cell lysis in primary CD4 T cells infected with and HIV-reporter virus. All experiments were methodologically correct, appropriate positive and negative controls were included in each experimental setting.

The observations made by the authors are discussed properly, in the context of current data and major conclusions are supported by presented data. The results are promising and it would be interesting to see how these therapeutic tools perform in preclinical settings; this will aid to better understand PKs of these constructs and in vivo efficacy. As mentioned by the authors those in vivo studies will be necessary to determine if the absence of Fc triggering and reduced neutralizing activity observed for BiCEs (compared to full parental NAbs) have an impact on in vivo efficacy. Other limitations of the present work are the lack of testing neutralizing activity of these constructs against a broader panel of viruses and a detailed molecular characterization of the BiCEs coupled to both target molecules (e.g., crystallography). These limitations might be mentioned in the discussion section.

Minor comments:
1. 3BNC117 was obtained from a patient infected with HIV clade B using a 2CC core (Hxb2) bait and 10-1074 from a clade A patient using a YU-2 bait; why did you pick YU-2 variant for neutralization tests and recognition on RAJI cells? Although no significant differences were observed with parental antibodies (3BNC117 and 10-1074) do you think that this could explain (at least in part) the lower performance of scFv3BNC117-Nb75 vs. ScFv10-1074-Nb75?
2. Figure 3E, last panel: why does C3 inhibitor have a minimal effect blocking rituximab CDC effect?
3. Is non-parametric Wilcoxon signed rank test proper for testing multiple groups?

Referee #3 (Comments on Novelty/Model System for Author):

In the manuscript entitled "Nanobody-mediated Complement Activation to Kill HIV-infected Cells" by Pedersen et al., the authors developed bispecific complement engagers (BiCE) which comprised of a nanobody recruiting the complement-initiating protein C1q, and single-chain variable fragments of broadly neutralizing antibodies (bNAbs) targeting the HIV-1 envelope protein. They showed that BiCEs mediate complement-dependent cytotoxicity of HIV-1 Env expressing Raji cells and HIV-1 infected primary CD4 T cells.
Overall, the data were clearly present, and the findings are of potential interesting. However, the data showed that the HIV neutralizing potency of the two novel anti-HIV BiCEs (scFV10-1074-Nb75 or scFV10-3BNC117-Nb75) were much lower than full length monoclonal bNAbs 10-1074 and 3BNC117 (Figure 2A). In addition, compared to bNAbs, the BiCEs did not show any better effect in mediating C3 deposition on HIV-1 Env expressing (Figure3C) or HIV-1 infected cells (Figure 4B). More importantly, the efficacy of BiCEs mediate complement-dependent cytotoxicity of HIV-1 Env expressing Raji cells or HIV-1 infected primary CD4 T cells was not better than bNAbs (Figure 3A and Figure 4D). Thus, the advantage to use BiCEs compared to bNAbs for HIV-1 treatment is questionable based on current data.
As the authors pointed out in the discussion section, the potential advantage of BiCEs over full-length bNAbs is its smaller size which may enable the increased tissue penetrance into the densely packed lymphoid tissues harboring HIV-infection. It is highly recommended to use relevant models such as HIV-infected humanized mice to prove whether BiCEs have better efficacy to mediated CDC of HIV-1 infected cells in lymphoid tissue in vivo.
The other potential advantage of BiCEs is its smaller size, which may allow competition for epitope sites in proximity such as the V3 loop and CD4 binding site of HIV Env. The manuscript could be significantly improved if the author can perform additional experiment to prove the hypothesis.

EMBO Molecular Medicine

Regarding manuscript entitled “Nanobody-mediated Complement Activation to Kill HIV-infected Cells”. Manuscript reference number: EMM-2022-16422.

We sincerely appreciate the time and effort that you and the reviewers devoted to providing thoughtful comments on our manuscript. The figures and text were clarified through these comments. In accordance with the suggestions by two of the reviewers, we also performed an *in vivo* experiment. Consequently, the manuscript now includes data from a pharmacokinetic study of BiCE in mice. Despite our best efforts, we were unable to examine the biodistribution of BiCE *in vivo*. We have tried to address the comments of the reviewers in our detailed point-by-point response – please see below. We believe this and the other changes to the manuscript has substantially improved the paper. Changes to the manuscript are highlighted in the compare-document included with the resubmission.

Sincerely,

Martin Tolstrup

***** Reviewer's comments *****

Referee #1 (Remarks for Author):

In this manuscript, Lange-Pedersen and colleagues report the creation and the validation of complement-activating anti-HIV-1 nanobodies. These new molecules attach to HIV-1 env on one side (using fragments from very well-known and potent antibodies) and to C1q on the other side. They call these molecules bispecific complement engager (BiCE). Then, they validate their capacity to mediate complement-mediated cytotoxicity in two different systems. First, using a cell line ectopically expressing HIV-1 Env and second using infected cells. They observe that their BiCE bind, trigger complement deposition and kill target cells. However, the outcome differs between the models, with either a rapid cell lysis in the cell line, or a delay killing in CD4 T cells. Pros: the BiCE platform is innovative and interesting, the authors successfully demonstrate the capacity of this platform to engage C1q and subsequently the complement cascade. Cons: the intrinsic resistance of HIV-1-infected cells limit the capacity of their compound to eliminate infected cells, it seems that BiCE performed just as well as antibodies to elicit complement, a few controls are missing. Overall, this is an interesting report, which deserves a few additional experiments and analysis prior to its publication.

We thank the reviewer for a favorable evaluation of the manuscript.

Specific comments:

-The various compounds are compared on a scale in ug/mL. Given their likely various molecular mass, it would be much more appropriate and infortive to compare them using molarity. It should be done prior to publication.

We appreciate the suggestion to refine the manuscript. We have changed the scale from µg/mL to nM in figure 2A.

- In figure 2A, additional compounds should be tested: the nanobody without the C1q binding part,

and antibody fab, the C1q bind part alone.. Overall, the anti-HIV-1 capacity of each individual part of BiCE deserves a more comprehensive assessment (of course the appropriate controls must be including). Minor point : the graph could be split into 2 graphs, according to the "backbone" either 3BNC117 or 10-1074, to facilitate reading.

We believe this is a valuable suggestion. Accordingly, we have tested the anti-HIV single chain variable fragment alone (scFv10-1074), the C1q binding nanobody alone (Nb75), and an irrelevant antibody (anti-tetanus antibody). The data from these additional control experiments are now included in Figure 2A. Additionally, we have split the figure into 3 graphs to facilitate reading.

- In the experiment with infected cells the choice of the viral strain is surprising and controls are lacking. Indeed, NL4.3 is known to be insensitive to 10-1074 neutralization and ADCC. Thus, it is expected to observe a lack of activity of the 10-1074-based BiCE. This is not the case in the author's data, which is possible, but additional controls are needed to support this claim. Indeed, there is neither an isotype control antibody nor an irrelevant BiCE included as controls in the experiment. It should be performed to ensure the relevance of the observed phenotype. Measuring the binding capacity of the BiCE at the surface of infected cells could also help interpret the data.

We value the suggestion and we have now included data testing the anti-HIV single chain variable fragment alone (scFv10-1074), the C1q binding nanobody alone (Nb75) and an irrelevant antibody (anti-tetanus antibody) in the *ex vivo* experiment. These are now included in Figure 4. However, we respectfully disagree that measuring the binding capacity of BiCE to the surface of infected cells is biologically relevant, as the expression of surface antigens on cells is dynamic and, particularly, HIV-1 infected cells will express different levels of surface antigens depending on cell state and level of viral replication.

We do agree with the reviewer that some papers showed that the HIV-1 NL4-3 strain is insensitive to neutralization and ADCC mediated by 10-1074. However, *von Bredow et al.* showed that cells infected with NL4-3 were sensitive to ADCC but not potent neutralization by 10-1074 (von Bredow *et al*, 2016). Importantly, we failed to describe that the NL4-3 strain used was modified to be CCR5 tropic (Münch *et al*, 2007). This modification has clearly changed the 10-1074 sensitivity. We have vigorously tested the sensitivity of the NL4-3-eGFP strain to both parental full-length 10-1074 and the scFv adaptation format in multiple cell-based assays.

-In figure 2 the bonding could be analyzed using the MFI, as the % of positive cells appears saturated in almost all conditions.

We appreciate the suggestion. However, we have chosen not to analyze the data using the MFI in Figure 2C because the detection antibody/protein in this assay cannot be compared directly. 10-1074 was detected using a secondary anti-IgG antibody whereas scFv10-1074 was detected using streptavidin-biotin linkage. The experiment was only intended to show that scFv can bind to HIV Env on the surface of cells. Therefore, we do not believe that the level of positive cells affects the interpretation of the data.

-Figure 1 C left, colors are uneasy to read

We apologize for making the colors in the graph uneasy to read. We have changed the colors to create greater contrast, and we hope this will aid reading.

-in the discussion, the sentence "Moreover, the smaller size of BiCEs may allow competition for epitope sites in proximity, such as the V3 loop and CD4 binding sites of HIV Env (Labrijn et al, 2003). "

is confusing, as 10-1074 and 3BNC117 target these epitopes and work very efficiently in combination.

We apologize for any confusion with this sentence. We agree with the reviewer that full-size 10-1074 and 3BNC117 work in combination in humans. We intended to write that the BiCEs may be more efficient in combination than the full-size antibody in combination. However, we have not tested that thoroughly. Consequently, we have decided to remove this sentence from the manuscript to avoid confusion and misleading text.

Referee #2 (Comments on Novelty/Model System for Author):

In general, the work is methodologically correct. These in-vitro and ex-vivo results are promising and it would be interesting to see how these therapeutic tools perform in preclinical settings; this will aid to better understand PKs/ biodistribution of these constructs and in vivo performance. Further, in vivo testing will inform if the absence of Fc triggering and reduced neutralizing activity observed for BiCEs (compared to full parental NAbs) have an impact on in vivo efficacy

Referee #2 (Remarks for Author):

In the present manuscript entitled "Nanobody-mediated Complement Activation to Kill HIV-infected Cells", Maria Lange Pedersen and colleagues describe the development and preliminary in vitro and ex vivo testing of a novel immunotherapy tool designed for HIV-1 treatment. This approach consists in a bispecific molecule directed to HIV-1 Env (single-chain variable fragments of broadly neutralizing antibody 3BNC117/ 10-1074) and the complement-initiating protein C1q (C1qNb75 nanobody). The constructs were tested for virus neutralization, HIV-Env recognition and complement activation in YU-2 Env expressing cell line. Furthermore, the authors tested the ability of anti-HIV BiCE to induce complement activation and cell lysis in primary CD4 T cells infected with and HIV-reporter virus. All experiments were methodologically correct, appropriate positive and negative controls were included in each experimental setting.

We thank the reviewer for the favorable comments about our work.

The observations made by the authors are discussed properly, in the context of current data and major conclusions are supported by presented data. The results are promising and it would be interesting to see how these therapeutic tools perform in preclinical settings; this will aid to better understand PKs of these constructs and in vivo efficacy. As mentioned by the authors those in vivo studies will be necessary to determine if the absence of Fc triggering and reduced neutralizing activity observed for BiCEs (compared to full parental NAbs) have an impact on in vivo efficacy. Other limitations of the present work are the lack of testing neutralizing activity of these constructs against a broader panel of viruses and a detailed molecular characterization of the BiCEs coupled to both target molecules (e.g., crystallography). These limitations might be mentioned in the discussion section.

We value the suggestion. However, we have chosen not to test the neutralizing activity against a broader panel of viruses as parental 10-1074 and 3BNC117 has been tested against various viruses previously (Mouquet *et al*, 2012; Scheid *et al*, 2011).

We thank you for suggesting a more detailed molecular characterization of the BiCE-antigen complexes. We did not perform crystallography of the BiCE-antigen complexes, as the epitopes of scFv10-1074 and scFv3BNC117 have already been described with the corresponding parental

antibodies (Mouquet *et al.*, 2012; Scheid *et al.*, 2011). Additionally, the Nb75-C1q complex has previously been described by crystallography by *Laursen et al.* (Laursen *et al*, 2020). We have added this information to the *Materials and Methods* section in the manuscript.

In accordance with suggestions from the reviewer, we have included a new experiment exploring the pharmacokinetics of BiCE to aid the *in vivo* understanding of these constructs. Briefly, scFv10-1074-Nb75 BiCE was administered intraperitoneally to mice. The serum concentration level of BiCE was frequently measured over a period of 46 hours. The mean serum concentration of scFv10-1074-Nb75 was 17.8 µg/mL after 30 minutes. After 2 hours, the serum level had decreased nearly 2 times. Indicating that scFv10-1074-Nb75 is cleared relatively fast from the blood. However, a low serum concentration of scFv10-1074-Nb75 was still observed after 46 hours. Data are shown in Supplementary Figure S3.

Minor comments:

1. 3BNC117 was obtained from a patient infected with HIV clade B using a 2CC core (Hxb2) bait and 10-1074 from a clade A patient using a YU-2 bait; why did you pick YU-2 variant for neutralization tests and recognition on RAJI cells? Although no significant differences were observed with parental antibodies (3BNC117 and 10-1074) do you think that this could explain (at least in part) the lower performance of scFv3BNC117-Nb75 vs. ScFv10-1074-Nb75?

We appreciate this thoughtful question. We used YU2 virus and YU2 expressing Raji cells, as the YU2 strain has been shown to be sensitive to both 3BNC117 and 10-1074. Further, we obtained the YU2 expressing Raji cells as a well-established read-out system of complement killing (Dufloo *et al*, 2020). It is possible that the lower performance of scFv3BNC117-Nb75 vs. scFv10-1074-Nb75 is partly explained by these differences. Similar small differences in parental antibodies (3BNC117 and 10-1074) ability to recruit complement and mediate CDC have previously been reported (Dufloo *et al.*, 2020).

2. Figure 3E, last panel: why does C3 inhibitor have a minimal effect blocking rituximab CDC effect?

This is a very good question. A mechanistic reason for the ongoing terminal complement pathway despite the effective inhibition of C3 can be explained by the strong activation of the classical pathway leading to dense C4b deposition on cells. This deposition of C4b (not only C3b) is capable of binding and priming C5 for the formation of a membrane attack complex leading to cell lysis, as recently reported by Mannes et al (Mannes *et al*, 2021). Therefore, potent activation of the classical pathway can bypass inhibition of C3 and activate C5, resulting in CDC. Rituximab is a very potent activator of the classical pathway through CD20 binding on B cells. In addition, CD20 is highly expressed on Raji cells, and we assume this will lead to a very strong complement activation compared to anti-HIV antibodies binding HIV Env on Raji cells. Consequently, a C3 inhibitor may not be fully capable of inactivating the classical complement cascade on rituximab treated Raji cells. However, we have not explored this mechanistic theory *in vitro.*

3. Is non-parametric Wilcoxon signed rank test proper for testing multiple groups?

We value this question. We have used a nonparametric test as we cannot assume the data are sampled from a Gaussian normal distribution given the n-value of the biological replicates. Our data are normalized to the no antibody treatment (value = 100%), and the median value in all treatment groups is calculated. This allows us to use the nonparametric Wilcoxon signed rank test which compares the median of each treatment group against a hypothetical median (in this case 100). We believe that this is the most suitable statistical test for our data.

Referee #3 (Comments on Novelty/Model System for Author):

In the manuscript entitled "Nanobody-mediated Complement Activation to Kill HIV-infected Cells" by Pedersen et al., the authors developed bispecific complement engagers (BiCE) which comprised of a nanobody recruiting the complement-initiating protein C1q, and single-chain variable fragments of broadly neutralizing antibodies (bNAbs) targeting the HIV-1 envelope protein. They showed that BiCEs mediate complement-dependent cytotoxicity of HIV-1 Env expressing Raji cells and HIV-1 infected primary CD4 T cells.

Overall, the data were clearly present, and the findings are of potential interesting. However, the data showed that the HIV neutralizing potency of the two novel anti-HIV BiCEs (scFV10-1074-Nb75 or scFV10-3BNC117-Nb75) were much lower than full length monoclonal bNAbs 10-1074 and 3BNC117 (Figure 2A). In addition, compared to bNAbs, the BiCEs did not show any better effect in mediating C3 deposition on HIV-1 Env expressing (Figure3C) or HIV-1 infected cells (Figure 4B). More importantly, the efficacy of BiCEs mediate complement-dependent cytotoxicity of HIV-1 Env expressing Raji cells or HIV-1 infected primary CD4 T cells was not better than bNAbs (Figure 3A and Figure 4D). Thus, the advantage to use BiCEs compared to bNAbs for HIV-1 treatment is questionable based on current data.

As the authors pointed out in the discussion section, the potential advantage of BiCEs over full-length bNAbs is its smaller size which may enable the increased tissue penetrance into the densely packed lymphoid tissues harboring HIV-infection. It is highly recommended to use relevant models such as HIV-infected humanized mice to prove whether BiCEs have better efficacy to mediated CDC of HIV-1 infected cells in lymphoid tissue in vivo.
The other potential advantage of BiCEs is its smaller size, which may allow competition for epitope sites in proximity such as the V3 loop and CD4 binding site of HIV Env. The manuscript could be significantly improved if the author can perform additional experiment to prove the hypothesis.

We appreciate your suggestion to refine the manuscript. In continuation of another comment from Referee 2 regarding this sentence, we have decided to remove this sentence from the manuscript to avoid any confusion and misleading text.

It has not been feasible for us to examine the functional efficacy of BiCE *in vivo* in HIV-infected humanized mice. However, we have explored the pharmacokinetic of the scFv10-1074-Nb75 BiCE *in vivo*. Briefly, scFv10-1074-Nb75 BiCE was administered intraperitoneally to mice. The serum concentration level of BiCE was frequently measured over a period of 46 hours. The mean serum concentration of scFv10-1074-Nb75 was 17.8 µg/mL after 30 minutes. After 2 hours, the serum level had decreased nearly 2 times. Indicating that scFv10-1074-Nb75 is cleared relatively fast from the blood. However, a low serum concentration of scFv10-1074-Nb75 was still observed after 46 hours. Data are shown in Supplementary Figure S3.

References:

Dufloo J, Guivel-Benhassine F, Buchrieser J, Lorin V, Grzelak L, Dupouy E, Mestrallet G, Bourdic K, Lambotte O, Mouquet H *et al* (2020) Anti-HIV-1 antibodies trigger non-lytic complement deposition on infected cells. *EMBO Rep* 21: e49351

Laursen NS, Pedersen DV, Gytz H, Zarantonello A, Bernth Jensen JM, Hansen AG, Thiel S, Andersen GR (2020) Functional and Structural Characterization of a Potent C1q Inhibitor Targeting the Classical Pathway of the Complement System. *Front Immunol* 11: 1504

Mannes M, Dopler A, Zolk O, Lang SJ, Halbgebauer R, Höchsmann B, Skerra A, Braun CK, Huber-Lang M, Schrezenmeier H *et al* (2021) Complement inhibition at the level of C3 or C5: mechanistic reasons for ongoing terminal pathway activity. *Blood* 137: 443-455

Mouquet H, Scharf L, Euler Z, Liu Y, Eden C, Scheid JF, Halper-Stromberg A, Gnanapragasam PN, Spencer DI, Seaman MS *et al* (2012) Complex-type N-glycan recognition by potent broadly neutralizing HIV antibodies. *Proc Natl Acad Sci U S A* 109: E3268-3277

Münch J, Rajan D, Schindler M, Specht A, Rücker E, Novembre FJ, Nerrienet E, Müller-Trutwin MC, Peeters M, Hahn BH *et al* (2007) Nef-mediated enhancement of virion infectivity and stimulation of viral replication are fundamental properties of primate lentiviruses. *J Virol* 81: 13852-13864

Scheid JF, Mouquet H, Ueberheide B, Diskin R, Klein F, Oliveira TY, Pietzsch J, Fenyo D, Abadir A, Velinzon K *et al* (2011) Sequence and structural convergence of broad and potent HIV antibodies that mimic CD4 binding. *Science* 333: 1633-1637

von Bredow B, Arias JF, Heyer LN, Moldt B, Le K, Robinson JE, Zolla-Pazner S, Burton DR, Evans DT (2016) Comparison of Antibody-Dependent Cell-Mediated Cytotoxicity and Virus Neutralization by HIV-1 Env-Specific Monoclonal Antibodies. *J Virol* 90: 6127-6139

24th Jan 2023

Dear Prof. Tolstrup,

Thank you for the submission of your revised manuscript to EMBO Molecular Medicine. I am pleased to inform you that we will be able to accept your manuscript pending the following final amendments:

1) Please address the referee #1 minor concerns.
2) Figures:
- Please upload individual, high-resolution files for the main and EV figures. For more information on figure presentation please check "Author Guidelines".
https://www.embopress.org/page/journal/17574684/authorguide#datapresentationformat
- In figures where n=2 (e.g. Figure 4B and D, Figure EV1) please show raw values from both measurements and remove error bars. When n=2 statistical analysis is not recommended and a justification for the use of the statistical test employed has to be provided. Please check our Author Guidelines:
https://www.embopress.org/page/journal/17574684/authorguide#statisticalanalysis
3) In the main manuscript file, please do the following:
- Correct/answer the track changes suggested by our data editors by working from the attached/uploaded document.
- Add callouts for Figure EV5.
- In M&M, provide the antibody dilutions that were used for each antibody.
- In M&M, statistical paragraph should reflect all information that you have filled in the Authors Checklist, especially regarding randomization, blinding, replication.
- Please rename "Competing Interest" to "Disclosure Statement & Competing Interests". We updated our journal's competing interests policy in January 2022 and request authors to consider both actual and perceived competing interests. Please review the policy https://www.embopress.org/competing-interests and update your competing interests if necessary.
- Author contributions: Please remove it from the manuscript and specify author contributions in our submission system. CRediT has replaced the traditional author contributions section because it offers a systematic machine-readable author contributions format that allows for more effective research assessment. You are encouraged to use the free text boxes beneath each contributing author's name to add specific details on the author's contribution. More information is available in our guide to authors:
https://www.embopress.org/page/journal/17574684/authorguide#authorshipguidelines
4) Appendix: Move supplementary methods to M&M section in the main manuscript text.
5) The Paper Explained: Please add it to the main manuscript.
6) Synopsis:
- Synopsis image: Please provide a striking image or visual abstract as a high-resolution jpeg file 550 px-wide x (250-400)-px high to illustrate your article.
- Please check your synopsis text and image before submission with your revised manuscript. Please be aware that in the proof stage minor corrections only are allowed (e.g., typos).
7) Source data: We encourage you to include the source data for figure panels that show essential data. Numerical data should be provided as individual .xls or .csv files (including a tab describing the data). For blots or microscopy, uncropped images should be submitted (using a zip archive if multiple images need to be supplied for one panel). Please check "Author Guidelines" for more information. https://www.embopress.org/page/journal/17574684/authorguide#sourcedata
8) For more information: This space should be used to list relevant web links for further consultation by our readers. Could you identify some relevant ones and provide such information as well? Some examples are patient associations, relevant databases, OMIM/proteins/genes links, author's websites, etc...
9) As part of the EMBO Publications transparent editorial process initiative (see our Editorial at http://embomolmed.embopress.org/content/2/9/329), EMBO Molecular Medicine will publish online a Review Process File (RPF) to accompany accepted manuscripts. This file will be published in conjunction with your paper and will include the anonymous referee reports, your point-by-point response and all pertinent correspondence relating to the manuscript. Let us know whether you agree with the publication of the RPF and as here, if you want to remove or not any figures from it prior to publication. Please note that the Authors checklist will be published at the end of the RPF.
10) Please provide a point-by-point letter INCLUDING my comments as well as the reviewer's reports and your detailed responses (as Word file).

I look forward to reading a new revised version of your manuscript as soon as possible.

Yours sincerely,

Zeljko Durdevic

Zeljko Durdevic
Editor
EMBO Molecular Medicine

***** Reviewer's comments *****

Referee #1 (Remarks for Author):

The author have answered my concerns, still the they should clarify this part of their response, which is rather unclear:

"We do agree with the reviewer that some papers showed that the HIV-1 NL4-3 strain is insensitive to neutralization and ADCC mediated by 10-1074. However, von Bredow et al. showed that cells infected with NL4-3 were sensitive to ADCC but not potent neutralization by 10-1074 (von Bredow et al, 2016). Importantly, we failed to describe that the NL4-3 strain used was modified to be CCR5 tropic (Münch et al, 2007). This modification has clearly changed the 10-1074 sensitivity. We have vigorously tested the sensitivity of the NL4-3-eGFP strain to both parental full-length 10-1074 and the scFv adaptation format in multiple cell-based assays."
Do they mean that NL43 is not a X4-tropic virus?

In the figure EV3, the x-axis should not be cut.

Referee #3 (Remarks for Author):

Is suitable for publication.

***** Reviewer's comments *****

Referee #1 (Remarks for Author):

The author have answered my concerns, still the they should clarify this part of their response, which is rather unclear:

"We do agree with the reviewer that some papers showed that the HIV-1 NL4-3 strain is insensitive to neutralization and ADCC mediated by 10-1074. However, von Bredow et al. showed that cells infected with NL4-3 were sensitive to ADCC but not potent neutralization by 10-1074 (von Bredow et al, 2016). Importantly, we failed to describe that the NL4-3 strain used was modified to be CCR5 tropic (Münch et al, 2007). This modification has clearly changed the 10-1074 sensitivity. We have vigorously tested the sensitivity of the NL4-3-eGFP strain to both parental full-length 10-1074 and the scFv adaptation format in multiple cell-based assays."

Do they mean that NL43 is not a X4-tropic virus?

We apologize that the previous answer did not clarify your question. The NL4-3 strain that we used in our laboratory was modified to be CCR5-tropic and not X4-tropic as the original NL4-3 strain is.

In the figure EV3, the x-axis should not be cut.

We appreciate the suggestion and we have now changed the x-axis.

Referee #3 (Remarks for Author):

Is suitable for publication.

We thank the reviewer for acknowledging our efforts to improve the manuscript for publication.

We are pleased to inform you that your manuscript is accepted for publication and is now being sent to our publisher to be included in the next available issue of EMBO Molecular Medicine.

EMBO Press Author Checklist

Corresponding Author Name: Martin Tolstrup
Journal Submitted to: EMBO Molecular Medicine
Manuscript Number: EMM-2022-16422

USEFUL LINKS FOR COMPLETING THIS FORM
The EMBO Journal - Author Guidelines
EMBO Reports - Author Guidelines
Molecular Systems Biology - Author Guidelines
EMBO Molecular Medicine - Author Guidelines

Reporting Checklist for Life Science Articles (updated January 2022)
This checklist is adapted from Materials Design Analysis Reporting (MDAR) Checklist for Authors. MDAR establishes a minimum set of requirements in transparent reporting in the life sciences (see Statement of Task: 10.31222/osf.io/9sm4x). Please follow the journal's guidelines in preparing your manuscript.
Please note that a copy of this checklist will be published alongside your article.

Abridged guidelines for figures
1. Data
The data shown in figures should satisfy the following conditions:
- the data were obtained and processed according to the field's best practice and are presented to reflect the results of the experiments in an accurate and unbiased manner.
- ideally, figure panels should include only measurements that are directly comparable to each other and obtained with the same assay.
- plots include clearly labeled error bars for independent experiments and sample sizes. Unless justified, error bars should not be shown for technical replicates.
- if n<5, the individual data points from each experiment should be plotted. Any statistical test employed should be justified.
- Source Data should be included to report the data underlying figures according to the guidelines set out in the authorship guidelines on Data Presentation.

2. Captions
Each figure caption should contain the following information, for each panel where they are relevant:
- a specification of the experimental system investigated (eg cell line, species name).
- the assay(s) and method(s) used to carry out the reported observations and measurements.
- an explicit mention of the biological and chemical entity(ies) that are being measured.
- an explicit mention of the biological and chemical entity(ies) that are altered/varied/perturbed in a controlled manner.
- the exact sample size (n) for each experimental group/condition, given as a number, not a range;
- a description of the sample collection allowing the reader to understand whether the samples represent technical or biological replicates (including how many animals, litters, cultures, etc.).
- a statement of how many times the experiment shown was independently replicated in the laboratory.
- definitions of statistical methods and measures:
  - common tests, such as t-test (please specify whether paired vs. unpaired), simple χ2 tests, Wilcoxon and Mann-Whitney tests, can be unambiguously identified by name only, but more complex techniques should be described in the methods section;
  - are tests one-sided or two-sided?
  - are there adjustments for multiple comparisons?
  - exact statistical test results, e.g., P values = x but not P values < x;
  - definition of 'center values' as median or average;
  - definition of error bars as s.d. or s.e.m.

Please complete ALL of the questions below.
Select "Not Applicable" only when the requested information is not relevant for your study.

Materials

Newly Created Materials	Information included in the manuscript?	In which section is the information available? (Reagents and Tools Table, Materials and Methods, Figures, Data Availability Section)
New materials and reagents need to be available; do any restrictions apply?	Yes	Materials and Methods

Antibodies	Information included in the manuscript?	In which section is the information available? (Reagents and Tools Table, Materials and Methods, Figures, Data Availability Section)
For **antibodies** provide the following information:<br>- Commercial antibodies: RRID (if possible) or supplier name, catalogue number and or/clone number<br>- Non-commercial: RRID or citation	Yes	Materials and Methods

DNA and RNA sequences	Information included in the manuscript?	In which section is the information available? (Reagents and Tools Table, Materials and Methods, Figures, Data Availability Section)
**Short novel DNA or RNA including primers, probes**: provide the sequences.	Not Applicable	

Cell materials	Information included in the manuscript?	In which section is the information available? (Reagents and Tools Table, Materials and Methods, Figures, Data Availability Section)
**Cell lines:** Provide species information, strain. Provide accession number in repository **OR** supplier name, catalog number, clone number, and/**OR** RRID.	Yes	Materials and Methods
**Primary cultures:** Provide species, strain, sex of origin, genetic modification status.	Yes	Materials and Methods
Report if the cell lines were recently **authenticated** (e.g., by STR profiling) and tested for mycoplasma contamination.	Yes	Materials and Methods

Experimental animals	Information included in the manuscript?	In which section is the information available? (Reagents and Tools Table, Materials and Methods, Figures, Data Availability Section)
**Laboratory animals or Model organisms:** Provide species, strain, sex, age, genetic modification status. Provide accession number in repository **OR** supplier name, catalog number, clone number, **OR** RRID.	Yes	Materials and Methods
**Animal observed in or captured from the field:** Provide species, sex, and age where possible.	Not Applicable	
Please detail **housing and husbandry conditions**.	Yes	Materials and Methods

Plants and microbes	Information included in the manuscript?	In which section is the information available? (Reagents and Tools Table, Materials and Methods, Figures, Data Availability Section)
**Plants:** provide species and strain, ecotype and cultivar where relevant, unique accession number if available, and source (including location for collected wild specimens).	Not Applicable	
**Microbes:** provide species and strain, unique accession number if available, and source.	Yes	Materials and Methods

Human research participants	Information included in the manuscript?	In which section is the information available? (Reagents and Tools Table, Materials and Methods, Figures, Data Availability Section)
If collected and within the bounds of privacy constraints report on age, sex and gender or ethnicity for all study participants.	Not Applicable	

Core facilities	Information included in the manuscript?	In which section is the information available? (Reagents and Tools Table, Materials and Methods, Figures, Data Availability Section)
If your work benefited from core facilities, was their service mentioned in the acknowledgments section?	Not Applicable	

Design

Study protocol	Information included in the manuscript?	In which section is the information available? (Reagents and Tools Table, Materials and Methods, Figures, Data Availability Section)
If study protocol has been **pre-registered, provide DOI in the manuscript**. For clinical trials, provide the trial registration number **OR** cite DOI.	Not Applicable	
Report the **clinical trial registration number** (at ClinicalTrials.gov or equivalent), where applicable.	Not Applicable	

Laboratory protocol	Information included in the manuscript?	In which section is the information available? (Reagents and Tools Table, Materials and Methods, Figures, Data Availability Section)
Provide DOI OR other citation details if **external detailed step-by-step protocols** are available.	Not Applicable	

Experimental study design and statistics	Information included in the manuscript?	In which section is the information available? (Reagents and Tools Table, Materials and Methods, Figures, Data Availability Section)
Include a statement about **sample size** estimate even if no statistical methods were used.	Yes	Materials and Methods
Were any steps taken to minimize the effects of subjective bias when allocating animals/samples to treatment (e.g. **randomization procedure**)? If yes, have they been described?	Yes	Materials and Methods
Include a statement about **blinding** even if no blinding was done.	Yes	Materials and Methods
Describe **inclusion/exclusion criteria** if samples or animals were excluded from the analysis. Were the criteria pre-established?<br><br>If sample or data points were omitted from analysis, report if this was due to attrition or intentional exclusion and provide justification.	Not Applicable	
For every figure, are **statistical tests** justified as appropriate? Do the data meet the assumptions of the tests (e.g., normal distribution)? Describe any methods used to assess it. Is there an estimate of variation within each group of data? Is the variance similar between the groups that are being statistically compared?	Yes	Materials and Methods

Sample definition and in-laboratory replication	Information included in the manuscript?	In which section is the information available? (Reagents and Tools Table, Materials and Methods, Figures, Data Availability Section)
In the figure legends: state number of times the experiment was **replicated** in laboratory.	Yes	Figures
In the figure legends: define whether data describe **technical or biological replicates**.	Yes	Figures

Ethics

Ethics	Information included in the manuscript?	In which section is the information available? (Reagents and Tools Table, Materials and Methods, Figures, Data Availability Section)
Studies involving **human participants**: State details of **authority granting ethics approval** (IRB or equivalent committee(s), provide reference number for approval.	Yes	Materials and Methods
Studies involving **human participants**: Include a statement confirming that **informed consent** was obtained from all subjects and that the experiments conformed to the principles set out in the WMA Declaration of Helsinki and the Department of Health and Human Services Belmont Report.	Yes	Materials and Methods
Studies involving **human participants:** For publication of **patient photos**, include a statement confirming that consent to publish was obtained.	Not Applicable	
Studies involving experimental **animals**: State details of **authority granting ethics approval** (IRB or equivalent committee(s), provide reference number for approval. Include a statement of compliance with ethical regulations.	Yes	Materials and Methods
Studies involving **specimen and field samples:** State if relevant **permits** obtained, provide details of authority approving study; if none were required, explain why.	Not Applicable	

Dual Use Research of Concern (DURC)	Information included in the manuscript?	In which section is the information available? (Reagents and Tools Table, Materials and Methods, Figures, Data Availability Section)
Could your study fall under dual use research restrictions? Please check biosecurity documents and list of **select agents and toxins** (CDC): https://www.selectagents.gov/sat/list.htm	Not Applicable	
If you used a select agent, is the security level of the lab appropriate and reported in the manuscript?	Not Applicable	
If a study is subject to dual use research of concern regulations, is the name of the **authority granting approval and reference number** for the regulatory approval provided in the manuscript?	Not Applicable	

Reporting

The MDAR framework recommends adoption of discipline-specific guidelines, established and endorsed through community initiatives. Journals have their own policy about requiring specific guidelines and recommendations to complement MDAR.

Adherence to community standards	Information included in the manuscript?	In which section is the information available? (Reagents and Tools Table, Materials and Methods, Figures, Data Availability Section)
State if relevant guidelines or checklists (e.g., **ICMJE, MIBBI, ARRIVE, PRISMA**) have been followed or provided.	Not Applicable	
For **tumor marker prognostic studies,** we recommend that you follow the **REMARK** reporting guidelines (see link list at top right). See author guidelines, under 'Reporting Guidelines'. Please confirm you have followed these guidelines.	Not Applicable	
For **phase II and III randomized controlled trials**, please refer to the **CONSORT** flow diagram (see link list at top right) and submit the CONSORT checklist (see link list at top right) with your submission. See author guidelines, under 'Reporting Guidelines'. Please confirm you have submitted this list.	Not Applicable	

Data Availability

Data availability	Information included in the manuscript?	In which section is the information available? (Reagents and Tools Table, Materials and Methods, Figures, Data Availability Section)
Have **primary datasets** been deposited according to the journal's guidelines (see 'Data Deposition' section) and the respective accession numbers provided in the Data Availability Section?	Not Applicable	
Were **human clinical and genomic datasets** deposited in a public access-controlled repository in accordance to ethical obligations to the patients and to the applicable consent agreement?	Not Applicable	
Are **computational models** that are central and integral to a study available without restrictions in a machine-readable form? Were the relevant accession numbers or links provided?	Not Applicable	
If publicly available data were reused, provide the respective **data citations in the reference list**.	Not Applicable	
